# Learner agency in a problem-based learning curriculum: A qualitative study on perspectives of undergraduate dental students

**DOI:** 10.1371/journal.pone.0346079

**Published:** 2026-03-26

**Authors:** Nidhi Gupta, Sruthi Sunil, Kamran Ali, Trine Fink, Xiangyun Du

**Affiliations:** 1 College of Dental Medicine, QU Health, Qatar University, Doha, Qatar; 2 Aalborg UNESCO PBL Centre, Department of Sustainability and Planning, Aalborg University, Aalborg, Denmark; 3 Department of Health Science and Technology, Aalborg University, Aalborg, Denmark; Ankara University Faculty of Medicine: Ankara Universitesi Tip Fakultesi, TÜRKIYE

## Abstract

Learner agency (LA), a student’s ability to take ownership of their learning and make purposeful decisions, is increasingly recognized as a critical competency in preparing adaptable healthcare professionals. While LA has been explored in general education, its development within dental education, particularly in the context of problem-based learning (PBL), remains underexplored. This qualitative study investigated undergraduate dental students’ perceptions of how a PBL curriculum supports the development of LA. Using purposive sampling, Year 2 and 3 students enrolled in a PBL-based dental program were invited to participate in focus group discussions conducted between September 2024 and March 2025. Four audio-recorded sessions were transcribed verbatim and analyzed thematically using a combined deductive-inductive approach with MAXQDA software. Twenty-two students participated in the study. Findings revealed that PBL supported key elements of LA, including enhanced motivation, self-efficacy, knowledge acquisition, goal setting, and reflective practices. Peer collaboration and facilitator guidance were identified as important enablers. However, challenges such as workload intensity, time management difficulties, and uneven peer participation were perceived as barriers to fully exercising agency. Overall, participants viewed PBL as a valuable foundation for fostering LA in dental education. Addressing structural and contextual barriers may further optimize its impact and better support the development of autonomous, self-regulated learners.

## Introduction

The dynamic and evolving nature of healthcare professions requires future practitioners to possess not only technical expertise but also the ability to adapt, make informed decisions, and take ownership of their professional development [1].These attributes are encapsulated in the concept of learner agency (LA), which refers to an individual’s ability and willingness to act with intention and purpose within a sociocultural context [2]. While LA has been explored in other fields, its application within health and dental education remains relatively underdeveloped. However, the relevance of LA in these contexts is increasingly recognized, as it emphasizes proactive engagement, autonomous decision-making, and the ability to navigate the complexities of clinical practice, competencies that are vital for cultivating confident, capable healthcare professionals [3].

Studies highlight that LA is shaped through continuous interactions between learners and their sociocultural environments, influenced by various contextual factors that either enhance or constrain their ability to engage independently in learning [2,4]. This aligns with student-centered educational models that emphasize autonomy, self-regulation, and active engagement, key factors in improving academic performance and fostering collaborative learning [5,6]. In health professions, cultivating LA is particularly crucial as it empowers students to take charge of their learning and respond to an ever-changing global healthcare landscape [3]. Therefore, learning environments that deliberately create space for learner participation, choice, and responsibility are likely to be central to how agency develops over time [3].

Problem-based learning (PBL) is one such environment. PBL is commonly described as a student-centered pedagogy in which learning is organized around authentic problems, small-group collaboration, and cycles of self-directed inquiry supported by facilitation rather than direct instruction [7]. These design features map closely onto core features of learner agency [5]. Specifically, PBL requires students to identify learning needs, propose explanations, negotiate learning goals with peers, and justify decisions through dialogue, activities that position learners as active contributors to the learning process rather than passive recipients [7]. In this sense, PBL does not only aim to transmit knowledge; it invites students to exercise agency through participation, decision-making, and accountability within a structured learning setting [5,8].

The LA–PBL relationship can be understood through a social cognitive lens. Bandura’s Social Cognitive Theory (SCT) conceptualizes human functioning through triadic reciprocal causation where personal factors, behaviors, and environmental conditions influence one another [9,10]. Within PBL, the learning environment (e.g., tutor facilitation, group norms, task design, and assessment signals) shapes students learning strategies thus making it a particularly suitable context for examining agency as an emergent, reciprocal process [11–13]. Complexity theory further strengthens this perspective by conceptualizing learning as emergent, nonlinear, and context dependent. Building on social cognitive principles, Morrison describes learning as a dynamic process shaped through continuous interaction between individuals and their environments [14]. Within PBL, learning develops through collaborative dialogue and engagement with uncertain problems, reflecting the complexity and contextual variability inherent in clinical practice [13,15]. This framing shows the active role of learners in co-constructing their learning environments rather than merely responding to them [5].

SCT also outlines the three interrelated dimensions of LA: intrapersonal, behavioral, and contextual [16]. The intrapersonal dimension encompasses self-efficacy, motivation, and knowledge acquisition, empowering students to take ownership of their learning [9,17]. The behavioral dimension involves active learning strategies such as goal setting, planning, and reflection, fostering autonomy and teamwork [18,19]. The contextual dimension highlights the role of social interactions, teacher support, and institutional structures in shaping agency, emphasizing the need for student-centered approaches [6,20]. Importantly, these dimensions are not independent; rather, they interact dynamically, making pedagogical models such as PBL particularly relevant because they intentionally configure peer interaction, facilitation, and responsibility structures that may support these dimensions [8].

Recent research on LA in medical education has examined its complexities within learning environments, highlighting both challenges and strategies for fostering agency [3,21–23]. Some scholars advocate for a shift toward systems agency, emphasizing the need for medical education to prepare learners to navigate and influence healthcare systems through clinical care, research, and lifelong learning [24]. Additionally, studies have explored the role of student representation in medical education, proposing models that balance student agency with institutional responsibility to enhance educational outcomes [22].

Despite growing interest in LA, there remains limited understanding of how it develops in dental education. Given that PBL is designed to promote self-directed inquiry and collaborative reasoning in the preclinical phase, it represents a plausible developmental context in which agency-related capabilities may be cultivated and later carried into clinical learning. To address this gap, the present qualitative study examined undergraduate dental students’ perceptions of how a PBL curriculum supports the development of LA and was guided by the research question: “How do undergraduate dental students perceive their development of learner agency within the context of a problem-based curriculum?”

## Methodology

### Study design

This study employed a qualitative research design with semi-structured focus groups to explore undergraduate dental students’ perceptions of LA development in a PBL curriculum. This approach captured the depth and complexity of student experiences in an interactive setting [25]. Focus groups facilitated structured yet flexible discussions, encouraging students to elaborate on their thoughts and uncover insights beyond rigid interviews. Their dynamic nature allowed participants to clarify viewpoints and build on each other’s responses [26]. Widely used in healthcare research, this method balanced structure and openness, enabling the exploration of topics significant to participants while allowing for new insights [27]. The study adhered to the Standards for Reporting Qualitative Research (SRQR) as outlined by O’Brien et al. (2014) [28], ensuring transparency and rigor in the design, analysis, and reporting of findings (see S1 Appendix).

### Study setting and context

This study was conducted at the newly established College of Dental Medicine, Qatar University, which offers a six-year integrated undergraduate curriculum leading to the Doctor of Dental Medicine degree. PBL is embedded throughout the program, with its most structured implementation occurring in Years 2 and 3. During these preclinical years, students participate in weekly PBL cycles built around clinical cases that simulate real-world dental scenarios and promote early development of clinical reasoning [29].

Each PBL cycle begins with a patient scenario through which students collaboratively identify learning objectives related to pathophysiology, diagnosis, investigations, and management. Students then engage in self-directed learning across the week and reconvene for a student-led synthesis session involving case presentations, group discussion, development of a concept map, and appraisal of a related research article. The PBL assessment rubric (see S2 Appendix) is provided to illustrate the competencies and attributes evaluated through this process.

As students progress through the program, PBL principles continue to shape learning. Core PBL practices such as self-directed inquiry, collaborative teamwork, effective communication, and the application of reasoning to authentic clinical cases remain embedded as the curriculum evolves [29]. In Year 4, these principles extend into project-based learning within Primary Care and Community Dentistry [30]. In Year 5, students participate in structured clinical decision-making exercises where they apply PBL-related processes to real patient cases. By Year 6 (internship year), students consolidate this learning through an exit case presentation that builds directly on their Year 5 case work, marking their transition from student to practicing clinician. Thus, while formal PBL sessions are concentrated in the early years, its underlying practices, self-directed inquiry, collaboration, case interpretation, and linking knowledge to patient care, remain integrated throughout all six years of the curriculum.

### Sampling technique and participants

A purposive sampling strategy was used to recruit undergraduate dental students with relevant experience in PBL, in accordance with qualitative research principles and guidelines for focus group recruitment [31]. The aim was not statistical representativeness but to obtain information-rich cases capable of providing insight into how learner agency develops within a PBL-based curriculum.

Students enrolled in Years 2 and 3 were invited to participate because these cohorts were actively engaged in the most structured phase of the PBL curriculum. Year 1 students were excluded due to limited exposure to PBL, and senior students (Years 4–6) were excluded as formal PBL sessions are less structured in the later years, and the study focused specifically on preclinical PBL experiences. An open invitation was emailed to all eligible Year 2 and Year 3 students (n = 65). Participation was voluntary. No selection criteria were applied beyond enrollment in Year 2 or 3 and prior exposure to at least one full PBL cycle. There were no academic performance or achievement-based inclusion criteria. Students who expressed interest were enrolled until the planned number of focus groups was achieved. Efforts were made to ensure diversity in gender and academic year representation across groups. Participants came from different PBL tutorial groups rather than a single intact PBL group, allowing for variation in tutor styles, peer dynamics, and group experiences. This approach minimized the influence of pre-existing group hierarchy and reduced conformity effects within discussions.

Qualitative research literature recommends conducting three to five focus groups, with 5–8 participants per group, to facilitate rich discussion while maintaining manageability [31]. In alignment with these recommendations, the study conducted four focus groups, a number considered adequate to reach thematic saturation. Each group comprised 5–6 participants, aiming to include diverse perspectives.

A total of 22 students (14 females and 8 males), aged between 19 and 22 years, participated in the study, representing approximately 34% of the eligible cohort of 65 (52 female,13 male) students. Participant characteristics are summarized in Table 1.

**Table 1:** Participant information.

Focus Group (FG) No.	Participant nos. (n)	Gender		Year
		Female (n)	Males (n)	
Group 1 *(FG1)*	5	2	3	2
Group 2 *(FG2)*	5	–	5	3
Group 3 *(FG3)*	6	6	–	3
Group 4 *(FG4)*	6	6	–	2
Total	22	14	8	–

### Data collection

Following ethical approval from Qatar University Institutional Review Board (QU IRB 210/2024-EA, dated 5 September 2024) and Aalborg University (IRB 2024-505-00408, dated 20 January 2025), data collection was done from 22^nd^ September to 27^th^ March 2025. An open invitation was emailed to students in Years 2 and 3, including a participant information sheet outlining the study’s objectives, scope, frequently asked questions and participant rights. The voluntary nature of participation and the right to withdraw at any time without penalty were emphasized. The principal investigator’s (NG) contact information was provided for any questions. Students who expressed interest contacted the principal investigator (NG) directly. Written informed consent was obtained prior to participation. Confidentiality, data handling procedures, and permission for audio recording were explained before the start of each session. Students were then allocated to focus groups based on availability, while ensuring representation from both academic years across groups. They were not grouped according to their existing PBL tutorial groups, thereby promoting open discussion and minimizing peer conformity related to prior group dynamics.

A topic guide (see S3 Appendix), developed from the literature, a recent scoping review [8] and aligned with the study’s conceptual framework, included both domain-specific and generic questions to explore the development of LA. All focus groups were facilitated by the principal investigator (NG), who possessed prior training and experience in conducting qualitative research [27].

Focus groups were conducted in English and each lasted approximately 50–60 minutes. Sessions were audio-recorded with participants’ consent, and supplementary handwritten notes were taken to capture key points and nonverbal cues. During the focus group sessions, participants were encouraged to elaborate on their experiences and perspectives regarding the questions in the topic guide and beyond [27].

### Data analysis

A combined analytic approach was used, integrating a theory-driven lens based on the three-dimensional framework introduced earlier with a bottom-up thematic analysis to capture participants’ perspectives of their LA within the study context. While the framework provided an initial orienting structure, the thematic analysis, conducted using MAXQDA (VERBI GmbH), allowed for coding, systematic data organization, and visualization, ensuring that both anticipated and emerging themes were represented in the findings [32].

Interview recordings were transcribed and reviewed line by line, with segments categorized according to participants’ descriptions, resulting in 348 initial codes. A codebook was developed and refined through iterative analysis of transcripts, audio recordings, and field notes to identify emerging themes. Data charting was conducted using Microsoft Excel to support systematic organization and comparison. Thematic analysis revealed patterns across all dimensions, with themes grounded in the data, supported by verbatim quotes, and centered on participants lived experiences.

To ensure credibility and trustworthiness, all focus group sessions were facilitated by the principal investigator, maintaining consistency in data collection. Additionally, a second investigator independently co-coded the transcripts using the thematic analysis framework that incorporated open coding techniques [33]. The co-coder was provided with the initial codebook for data extraction, and interrater reliability was calculated on 10% of the transcripts, yielding a high agreement rate of 85%. Both coders worked independently, with the second coder blinded to the first author’s input to reduce bias. An independent qualitative expert reviewed the thematic structure, leading to minor refinements, and member checking was conducted with five participants to confirm the accuracy of the interpretations. Preliminary findings were also shared with selected participants to confirm the accuracy of interpretations.

Credibility and reflexivity were further enhanced through a collaborative approach to data collection and analysis. While the first, second and third authors, affiliated with the host institution, brought insider perspectives, other co-authors contributed external viewpoints. The team engaged in continuous dialogue and joint interpretation of the data, critically reflecting on emerging themes and challenging assumptions. Reflexive practices, such as maintaining reflective journals and documenting analytic decisions, were employed throughout to enhance transparency and trustworthiness. An illustrative example of data analysis, including the development of themes through deductive and inductive coding, is presented in S4 appendix.

## Results

This study explored how undergraduate dental students perceived the development of LA in a PBL-based curriculum. The analysis revealed three interrelated dimensions of agency: intrapersonal, behavioral, and contextual. Within these dimensions, multiple sub-themes emerged that reflected both enabling conditions and challenges influencing how students enacted agency across learning environments. The analytic process combined deductive coding based on the study’s three-dimensional framework with inductive theme refinement within each dimension [9,10]. This approach ensured alignment with agency theory while allowing participant-driven meanings to surface. Table 2 provides an overview of the thematic structure, including dimensions, sub-themes and the proportion of participants referencing each theme (used only to illustrate emphasis within the qualitative dataset).

**Table 2:** Thematic coding with interpretive descriptions.

Dimension	Sub-theme	Participants referencing theme (n, %)	Interpretive Summary
Intrapersonal	Motivation	20 (~91%)	Real-life PBL cases enhanced curiosity and intrinsic motivation.
	Self-efficacy	15 (~68%)	Participants felt increasingly confident engaging in learning tasks and challenges in PBL.
	Knowledge acquisition	11 (~50%)	PBL supported structured understanding and deeper independent learning.
		8 (~36%)	Sequential case discussions facilitated progressive knowledge building.
	Communication	19 (~86%)	PBL strengthened interpersonal and academic communication skills.
		8 (~36%)	Repeated presentation cycles improved clarity and structure.
	Career perspective	10 (~45%)	Participants reported developing a clearer sense of emerging professional identity.
Behavioral	Goal setting	15 (~68%)	Students frequently set short-term, achievable goals through PBL.
		6 (~30%)	Some shifted toward longer-term, self-improvement-oriented goals.
		7 (~35%)	Others struggled with goal maintenance due to workload and pace.
	Planning and time management	16 (~73%)	Time demands posed challenges; some improved planning through PBL structure.
		5 (~20%)	PBL routines promoted better organization for a subset of participants.
	Reflection and monitoring	18 (~80%)	Participants used feedback and reflection to refine strategies and monitor progress.
Contextual	Team dynamics	18 (~82%)	Collaborative discussions shaped understanding and enhanced reasoning.
	Facilitator support	20 (~90%)	Facilitators provided valued structure, guidance, and reassurance.
	Institutional support	10 (~45%)	Orientation visits and institutional resources supported agency development.

### Dimension 1: Intrapersonal aspects

The intrapersonal dimension encompassed participants’ beliefs, motivations, and developing competencies that shaped their sense of agency within the PBL curriculum. Five sub-themes were identified: motivation, self-efficacy, knowledge acquisition, communication, and career perspective.

#### Motivation – engaging in clinical problem-solving.

Across focus groups, majority of the participants consistently described motivation as a cornerstone of their LA. They viewed PBL as an approach that made learning purposeful, largely because cases resembled authentic clinical challenges. Participants repeatedly emphasized that working through realistic scenarios enhanced their motivation and gave purpose to their studies. As one participant stated, “*Understanding how the case-based scenarios in PBL apply to real-life practice, for example, learning about managing diabetic patients in dental settings, motivates me and makes studying more meaningful.” (FG2; participant 5).*

For many, PBL also shifted their studying method, where learning became a self-driven process rather than merely a response to assessments. As they moved from passive to active learning they began to internalize the value of their education: *“With PBL, it felt like I was learning for myself rather than just for an assessment.” (FG1; participant 1).*

While grades and feedback certainly mattered to participants, their experiences were far from uniform. For some, assessment pushed them to prepare well and stay engaged, *“Grades motivate me to prepare better, and I like knowing how my effort translates into results.” (FG1; participant5).* For others, the pressure of performing, especially when expectations felt unclear, sometimes dampened their enthusiasm. Several participants noted that it wasn’t “*the grade itself but how feedback was given that made the real difference” (FG4; participant 5)*. When feedback was clear, constructive, and delivered in a supportive way, “*it strengthened their motivation”(FG2; participant 2)* and helped them stay committed to learning.

#### Developing confidence- from uncertainty to self-belief.

After motivation, self-efficacy emerged as another key component of the intrapersonal dimension. Participants described their journey in PBL as one that often began with uncertainty but gradually evolved into a stronger belief in their ability to learn independently. Many recalled feeling overwhelmed during the initial sessions, yet over time, several noted that working through PBL projects not only boosted their confidence but also deepened their understanding of professional knowledge. As participants stated, *“At first, I felt really overwhelmed with the PBL process. Initially, I thought, ‘We’ll just learn these things in lectures, so why go through this process of finding learning objectives?’ But as the weeks went on, I realized that finding these objectives helped with my critical thinking skills.” (FG1; participant 1)*

*“I realized that the knowledge gained in the course can be directly applied to my clinical cases. I feel that by the time I complete my training, I will have a thorough understanding of the concepts and their practical application in patient care.” (FG3; participant 1)*

#### Knowledge acquisition- building understanding through inquiry.

Participants described PBL as a powerful catalyst for deeper understanding, helping them towards more meaningful engagement with course material. About half of them noted that PBL supported independent learning and “*encouraged them to organize information in a clearer, more systematic way” (FG2; participant 5)*. Several also valued how each session built progressively on the last, mirroring the unfolding of real clinical cases and prompting them to think critically, make connections, and actively construct knowledge rather than simply receiving it. As one participant very aptly said, *“Each PBL trigger starts with minimal information, and as we progress, our knowledge and understanding develops markedly. It starts wiring your brain to think about the patient’s journey and even as a student, we tend to think like dentists.” (FG1; participant1)*

Some other participants reflected on their earlier experiences with lecture-based learning, where information was largely delivered to them and their role was mostly passive. In comparison, PBL required them to engage actively exploring problems, debating ideas with peers, and piecing together their own understanding. One of them expressed, *“In a traditional lecture, they usually tell you what you need to learn and where you’re supposed to be, almost putting you in a box. But in PBL, you’re allowed to think outside the box, explore different ideas, and then circle back to the learning objectives.” (FG2; participant 5)*

#### Communication skills.

Participants unanimously highlighted communication as one of the most significant strengths they gained from PBL. Almost one third of them felt that regular presentations, collaborative discussions, and the expectation to articulate complex ideas enhanced interpersonal skills. As one participant reflected, *“We present our findings at the end of the week, which helps build presentation skills. As future dentists, we’ll need to present patient cases, progress, and prognosis concisely.” (FG4; participant 5).*

They also mentioned that PBL supported the development of interpersonal communication, an essential skill often overlooked in formal education but critical in clinical practice. One participant articulated this very well, *“Patients don’t always understand technical details, but they notice how they’re treated. Unfortunately, communication isn’t something universities focus on adequately; it’s a skill we develop personally. That’s where PBL sessions are helpful, they put us in social settings and improve our communication skills.” (FG2; participant 3).*

#### Evolving career perspective.

The PBL environment played a significant role in shaping the participants’ early perceptions of their future roles in the dental profession. As participants progressed through the curriculum, almost half of them began to see themselves not just as learners but as emerging professionals within the dental field. As one stated, *“When I talk to my friends now, I feel much more like a dentist. I can engage in conversations about dentistry and feel like I’m becoming part of the dental community” (FG2; participant 5).* Another participant described how the “*hands-on nature of training, combined with the structured problem-solving approach in PBL helped them develop a clearer sense of their professional trajectory” (FG3; participant 4).*

For others, PBL fostered a mindset aligned with professional practice by emphasizing critical thinking and patient-centered care. As one participant reflected, *“PBL helps us think like professionals. Instead of just reading about a disease, we learn how to approach a case, ask questions, and think of the patient’s needs. It prepares us for how dentists should think.” (FG4; participant 1).* Some expressed the need for stronger dental specificity within cases: *“PBL contributes to my future profession as a dentist. However, I wish it were more dentistry focused. While medical content is important, we’d like to learn more about dentistry” (FG3; participant 6).* In addition, participants reflected on how their “*career aspirations evolved through the PBL experience, from focusing solely on academic performance to considering broader professional goals, including specialization and research*” *(FG2; participant 4)*

### Dimension 2: Behavioral aspects

Participants described the behavioral dimension of LA as being shaped through the rhythm and demands of the PBL cycle. Across groups, they emphasized that the weekly structure pushed them to set goals, plan their work, and reflect on their progress although they differed in how successfully they managed these expectations. For some, PBL created a sense of routine and accountability; for others, the pace and workload made self-regulation more challenging. One participant captured this tension bluntly: *“PBL helps with problem-solving and communication, but the workload makes self-regulation challenging.” (FG3, participant 5)*

#### Goal setting.

Many participants felt that the time-bound nature of each PBL cycle compelled them to set short-term, achievable academic goals. They contrasted this with traditional lecture-based learning, where engagement could be delayed until exams approached. In PBL, the need to synthesize and present learning by the end of the week created a sense of urgency and helped them structure what mattered most. As one participant explained: *“With traditional methods, we might cover a topic over a month before our first assessment. With PBL, we have to master the topic of the week to present it by Thursday, so it’s much better.” (FG1, participant 3)*

Several participants described a shift from externally driven goals toward personal improvement, explaining that PBL motivated them to measure progress against their own standards rather than competing with peers. One participant shared:

*“PBL has helped me focus on self-improvement… I used to compare myself with others, but now I’m trying to be better than I was the day before.” (FG2, participant 1)*

However, not all participants found goal setting easy. Some described struggling to keep up with the fast pace of weekly tasks, which sometimes led to last-minute preparation and reactive learning rather than intentional planning: *“I struggle to keep up with my learning goals because time management and stress are ongoing challenges.” (FG4, participant 2)*

#### Planning, time management and adaptability.

Participants discussed time management as both a support and a strain within the PBL curriculum. Those who felt PBL improved their organization appreciated the focused weekly structure, which helped them prioritize and sequence their learning: *“In PBL, we focus on one topic each week, which helps us stay on track and link topics logically.” (FG1, participant 4)*

For these participants, PBL provided a scaffold for planning ahead and adapting to unexpected demands, skills they viewed as essential preparation for clinical training. One participant described how weekly accountability shaped stronger habits: *“Since we have to stay on track, PBL teaches us how to plan ahead. When unexpected events come up, having a good plan helps minimize disruptions.” (FG2, participant 1)*

Others found the demands overwhelming. They described working in “emergency mode,” trying to complete tasks as deadlines approached rather than following a structured plan. As one student put it: *“I manage with an emergency mechanism, doing as much as I can before the deadline.” (FG3, participant 5)*

#### Monitoring progress and reflection.

Reflection played a vital role in how participants monitored their progress. Many described using PBL sessions to evaluate what they understood, identify gaps, and learn by observing peers. The collaborative nature of PBL encouraged them to examine their strengths and weaknesses, not only through self-reflection but also through peer comparison and feedback. One participant shared: *“PBL encourages us to reflect on our own abilities and learn from others.” (FG4; participant 2)*

Participants valued opportunities to assess both their own work and their peers,’ describing this process as central to improving understanding over time. Presentations and group discussions served as informal checkpoints that helped them adjust and refine their learning strategies: *“Most of the time we evaluate each other’s work and give feedback. This helps us because we then reflect on our own work and try to improve it.” (FG4; participant 1)*

### Dimension 3: Contextual aspects

The contextual dimension captured the relational and institutional conditions that played a role in participants’ enactment of agency such as team dynamics, facilitator and institutional support.

#### Team dynamics.

Peer interactions formed a central part of participants’ PBL experience. Many participants described their groups as sources of both academic and emotional support, noting that collaborative work expanded their thinking and helped them navigate the demands of weekly tasks. Several students explained that their PBL groups became informal learning communities beyond the classroom, sustaining shared responsibility and a sense of belonging. One participant described their group chats as a key resource:

*“We use our PBL group chats to share resources and ask each other questions. Before exams, we even send self-made questions to quiz one another. It really helps us see our weak points and revise our mistakes.” (FG4; participant 1)*

Others emphasized the value of dialogue, explaining that hearing alternative viewpoints strengthened their reasoning and deepened their understanding: *“Learning with others is invaluable. It offers diverse perspectives and helps solidify our understanding.” (FG4; participant 3)*

For many, working closely in teams also nurtured the interpersonal qualities they associated with future professional practice. As one participant noted:

*“Working as a team is essential especially in healthcare since in the future, we will collaborate with various professionals to provide comprehensive care.” (FG4; participant 2)*

However, not all experiences were uniformly positive. A few participants noted challenges such as *“unequal participation or occasional group tension, which at times disrupted the learning process”(FG3; participant 4).*

#### Facilitator support.

Across focus groups, participants described facilitators as pivotal to shaping the tone and productivity of PBL sessions. Many appreciated how facilitators helped clarify expectations, guided discussions, and provided reassurance when the material felt overwhelming. Participants often noted that timely feedback and access to facilitators strengthened both their confidence and their understanding. One participant explained:

*“The dental staff really helps. They’re considerate and supportive, which boosts my confidence. The sessions feel well-rounded, and the theory connects to the practical work, making it easier to apply what we learn.” (FG4; participant 2)*

Facilitators were also credited with helping groups maintain focus without taking over the learning process. Participants valued this balance, describing it as essential to keeping discussions meaningful while still preserving student autonomy:

*“Although we assign a leader in our PBL group, the facilitator guides the conversation and keeps us on track. They help us focus on the important parts without giving away the learning objectives.” (FG1; participant 2)*

#### Support from institution.

Participants also reflected on the broader structures of the program, identifying several institutional features that supported their development. Well-organized learning weeks, alignment between PBL and lectures, and the opportunity to practice clinical skills were frequently mentioned as important foundations for building confidence:

*“Our college does a great job by giving us multiple opportunities to practice skills, which builds confidence over time.” (FG3; participant 6)*

However, participants also pointed out areas where institutional support felt limited. Some noted that while academic and mental health resources existed, they were not always easy to access or clearly communicated. Others expressed a desire for their feedback to be taken more seriously in shaping the learning environment:

*“I feel that the institution could take our feedback more seriously and make changes that create a more effective learning environment.” (FG3; participant 1)*

At times structural pressures, especially workload, time constraints, and grading expectations created strain. Some participants described “*feeling fatigued or disengaged during high-stress periods” (FG2; participant 5)*, which they felt limited their ability to approach learning intentionally or maintain consistent self-regulation.

## Discussion

This research contributes to the limited body of qualitative literature examining LA in undergraduate dental education through the lens of a problem-based curriculum. Drawing on Bandura’s SCT, which emphasizes the triadic interaction between personal, behavioral, and environmental factors, and informed by Complexity Theory, this study provides insights into how intrapersonal beliefs, self-regulatory behaviors, and contextual dynamics interact to shape students’ agentic development [9,14,16]. The findings illuminate the dynamic interplay among intrapersonal, behavioral, and contextual dimensions in shaping students’ evolving sense of agency as self-directed, motivated and reflective learners preparing for clinical practice.

In this study, motivation emerged as a pivotal factor, particularly when participants encountered real-life clinical challenges in PBL. Participants described how working on real-life patient scenarios gave purpose to their learning and enhanced intrinsic motivation. These findings support Ryan and Deci’s (2000) theory that autonomy and relevance enhance intrinsic motivation [17]. While some participants continued to value extrinsic motivators such as grades, many transitioned toward more intrinsic goals rooted in personal growth and professional relevance. However, concerns about performance anxiety and assessment pressure suggest that assessment design and feedback quality play critical roles in either enabling or constraining motivation [34].

Closely linked to motivation was the development of self-efficacy. As students engaged iteratively with PBL tasks, their confidence in managing clinical content and learning processes increased. Bandura (2006) identifies mastery experiences as essential to building self-efficacy, which in turn supports self-directed learning and goal pursuit [9]. Participants’ strategies reflected core aspects of self-directed and metacognitive learning. Their ability to integrate new knowledge with prior understanding aligns with Bagga’s (2024) findings on reflective knowledge construction [35]. Their evolving self-awareness and ability to monitor their learning highlight the importance of cultivating metacognitive skills to support self-directed learning and agency. These varied learner experiences may be used to inform future strategies to facilitate the enactment of agency in students and to cater to diverse learning styles.

Participants evolving career perspectives reflected a shift from academic compliance to professional alignment. As they progressed through the curriculum, they began to envision themselves as future dental professionals, not just learners. This aligns with research showing that active knowledge construction in socially authentic contexts strengthens students’ sense of purpose and direction [13,36]. Yet several participants felt that PBL scenarios needed stronger dentistry-specific content to deepen this emerging career focus. Their feedback highlights a persistent challenge in integrated curricula that while broad medical cases promote interdisciplinarity, they can unintentionally dilute dental relevance, a concern echoed in prior research [5].

The structure of the PBL process encouraged participants to focus on short-term academic goals, such as setting weekly objectives and organizing study schedules. This emphasis on immediate, task-specific learning was supported by the time-bound nature of PBL cycles and aligns with Zimmerman’s (2002) model of SRL [37–39]. However, the findings also reveal variability in SRL proficiency. Some participants relied on last-minute strategies to meet deadlines, indicating that without adequate support, the autonomy embedded in PBL can become overwhelming. These findings align with Ali et al. (2023), who argue that while PBL fosters autonomy, structured instructor support is crucial to scaffold the development of SRL [37]. In the context of PBL, this also points out to how the demands of weekly tasks and self-directed preparation can present challenges. It also reflects the interconnected nature of SRL components such as goal setting, time management, and stress regulation. A breakdown in any one of these areas, such as failing to plan or feeling overwhelmed, can trigger a chain reaction that hinders overall engagement and learning effectiveness within the PBL framework.

Reflection further emerged as another key mechanism through which participants enacted agency. Feedback, both peer and facilitator-driven, prompted self-assessment and goal adjustment. These findings reinforce the importance of guided reflection in promoting intentional learning behaviors [40,41]. Expanding opportunities for structured reflection beyond PBL, such as through portfolios or journaling, could further enhance resilience and professional development [42].

Participants also discussed the development of both communication and presentation skills through their engagement in PBL. Weekly presentations enhanced clinical reasoning and public speaking, while collaborative discussions supported the growth of interpersonal communication, an area often underemphasized in traditional dental curricula yet critical to clinical practice. This dual skill development aligns with previous research indicating that PBL fosters both cognitive and interpersonal competencies by engaging students in socially interactive, learner-centered environments where such skills are continuously developed and refined [43].

Peer collaboration was another important contextual factor. Participants noted the benefits of mutual support and knowledge sharing. However, the informal and occasionally unstructured nature of group interactions was seen to limit individual accountability. As such, alternative models such as team-based learning, which provide structured interdependence and individual accountability, may complement PBL and enhance both teamwork and learning outcomes [44,45]. Facilitators played a critical role in balancing autonomy with appropriate guidance. Participants valued facilitators who provided timely feedback and kept discussions focused without dominating them. However, concerns about inconsistent facilitation echoed findings from other studies, emphasizing the need for faculty development to ensure equitable support across groups [5,46–48].

At the institutional level, participants acknowledged the college’s role in structuring PBL weeks and providing opportunities for skill development. Participants mentioned their wishes for adopting a pass/fail grading system, a change that has been associated with reduced stress and improved learning outcomes in health education [49,50]. These findings reflect Alrahlah’s (2016) concern that institutional demands can counteract the benefits of PBL, suggesting that systemic changes in assessment and workload distribution are necessary to sustain LA [43].

The results of this study offer several implications for enhancing LA in dental education. For students, developing awareness of their own agency through reflective tools, goal-setting workshops, and feedback literacy training can empower them to take proactive ownership of their learning. For educators, professional development programs should emphasize the role of facilitators as coaches who scaffold SRL and identity formation without diminishing autonomy. Educators must also recognize variability in student readiness for agency and adapt support accordingly. At the institutional level, adopting alternative assessments and facilitating guided reflection may help create a learning environment where agency is nurtured. Ultimately, aligning curriculum, pedagogy, and institutional culture with the principles of LA can support the development of confident, competent, and adaptive dental professionals.

A few limitations should be acknowledged. Firstly, the study focused on participants in the early stages of their dental education. As a result, it may not fully capture how LA evolves as students transition into more advanced clinical phases and engage in direct patient care. Longitudinal research that follows students throughout their study journey would offer deeper insights into the changing nature of LA over time.

Secondly, this study was conducted within a single institutional context, which limits the generalizability of the results. Institutional culture, curriculum design, and pedagogical approaches differ widely across dental schools, potentially shaping LA in unique ways. Future studies incorporating multiple institutions with varied educational contexts would enhance external validity and provide a broader perspective on LA in undergraduate dental education.

Thirdly, the empirical data derived was solely from focus group discussions. Although credibility was supported through triangulation, reflective memo writing, and team-based analysis, the trustworthiness of the findings could be further strengthened by incorporating multiple data sources. These might include classroom or team-based session observations, individual interviews, or quantitative measures.

Finally, the participant sample had a gender imbalance, with only 13 male students included (three in Year 2 and ten in Year 3). This skewed distribution may have influenced the diversity of perspectives captured, particularly if gender-related factors affect how LA is perceived and enacted. Future research should strive for greater demographic balance to ensure a more inclusive representation of learner experiences.

## Conclusion

This study demonstrates that LA is a dynamic, multidimensional construct emerging through the interaction of internal beliefs, purposeful behaviors, and supportive contexts. PBL provides a conducive environment for LA to flourish, enabling students to become more motivated, self-efficacious, reflective, and professionally engaged. However, the realization of this potential depends on intentional curriculum design, balanced assessment practices, faculty support, and institutional policies that prioritize student autonomy and agency.

## Supporting information

S1 Appendix SRQR checklist. (DOCX)

S2 Appendix PBL rubric. (PDF)

S3 Appendix Topic guide. (DOCX)

S4 Appendix Coding scheme with sample themes and data. (DOCX)
