# Supplementary material for: Learner agency in a problem-based learning curriculum: A qualitative study on perspectives of undergraduate dental students
Source: PLoS One. 2026 Mar 26;21(3):e0346079. doi: 10.1371/journal.pone.0346079 (PMC13020853; doi:10.1371/journal.pone.0346079)
Supplement: S1 Appendix — (DOCX) [file pone.0346079.s001.docx]

| **Index** | **Topic** | **Page(s)** |
| --- | --- | --- |
|  | **Title and Abstract** |  |
| 1 | Title | 1 |
| 2 | Abstract | 2 |
|  | **Introduction** |  |
| 3 | Problem formulation | 3-5 |
| 4 | Purpose or research question | 5-6 |
|  | **Methods** |  |
| 5 | Qualitative approach and research paradigm | 6-8 |
| 6 | Researcher characteristics and reflexivity | 11-12 |
| 7 | Context | 6-7 |
| 8 | Sampling strategy | 8-9 |
| 9 | Ethical issues pertaining to human subjects | 8-9 |
| 10 | Data collection methods | 10-11 |
| 11 | Data collection instruments and technologies | 10-11 |
| 12 | Units of study | 11 |
| 13 | Data processing | 10-11 |
| 14 | Data analysis | 11-12 |
| 15 | Techniques to enhance trustworthiness | 10-11 |
|  | **Results** |  |
| 16 | Synthesis and interpretation | 12-24 |
| 17 | Links to empirical data | 12 |
|  | **Discussion** |  |
| 18 | Integration with prior work, implications, transferability, and contribution(s) to the field. | 25-29 |
| 19 | Limitations | 29 |
|  | **Other** |  |
| 20 | Conflicts of interest | Provided in submission system |
| 21 | Funding |  |

**S1 Appendix: Standards for Reporting Qualitative Research (SRQR) Checklist**

**Reference:**

O'Brien BC, Harris IB, Beckman TJ, Reed DA, Cook DA. Standards for reporting qualitative research: a synthesis of recommendations. Academic Medicine, Vol. 89, No. 9 / Sept 2014 DOI: 10.1097/ACM.0000000000000388
