# Supplementary material for: Learner agency in a problem-based learning curriculum: A qualitative study on perspectives of undergraduate dental students
Source: PLoS One. 2026 Mar 26;21(3):e0346079. doi: 10.1371/journal.pone.0346079 (PMC13020853; doi:10.1371/journal.pone.0346079)
Supplement: S2 Appendix — (PDF) [file pone.0346079.s002.pdf]

كلية طب الأسنان
College of Dental Medicine
QATAR UNIVERSITY جامعة قطر
Member of HEALTH الصحة عضوفي

PBL RUBRIC

Professionalism			Scientific Knowledge			Research	Communication skills	Engagement and Team working	Leadership
**Responds positively to constructive criticism.**	**Demonstrates effective time management.**	**Recognizes the learning needs of colleagues.**	**Identifies learning objectives**	**Demonstrates knowledge of relevant medical conditions and their dental implications**	**Organizes and presents information effectively (concept map)**	**Recognizes the importance of evidence-based practice**	**Communicates effectively with other health care professionals**	**Engages with colleagues effectively to demonstrate team working skills**	**Engages in leadership and mentor/mentee activities within an oral health care team**
Above standards (Positive response) (1)	Above standards (Time managed well) (1)	Above standards (Good understanding) (1)	Above standards (Identifies all objectives) (1)	Above standards (Good understanding) (1)	Above standards (Done correctly) (1)	Above standards (Good understanding) (1)	Above standards (Good communication) (1)	Above standards (Effective engagement and team working skills) (1)	Above standards (Effective engagement) (1)
Meets standards (Moderately positive response) (0.75)	Meets standards (Exceeds time by quarter) (0.75)	Meets standards (Moderately understands) (0.75)	Meets standards (Moderately identifies Objectives) (0.75)	Meets standards (Moderately understands) (0.75)	Meets standar ds (Minor errors) (0.75)	Meets standards (Moderately understands) (0.75)	Meets standards (Moderate communication) (0.75)	Meets standards (Moderate engagement and team working skills) (0.75)	Meets standards (Moderate engagement) (0.75)
Borderline (Partially positive response) (0.5)	Borderline (Exceeds time limit by half or more) (0.5)	Borderline (Partially understands) (0.5)	Borderline (Partially identifies) (0.5)	Borderline (Partially understands) (0.5)	Borderline (More than 2 errors) (0.5)	Borderline (Partially understands) (0.5)	Borderline (Partial communication) (0.5)	Borderline (Partial engagement/ team working skills) (0.5)	Borderline (Partial engagement) (0.5)
Below standard (Negative response) (0)	Below standard (No concept of time limit) (0)	Below standard (Does not understand) (0)	Below standard (Does not identify) (0)	Below standard (Does not understand) (0)	Below standard (Major errors) (0)	Below standard (Does not understand) (0)	Below standard (Poor communication) (0)	Below standard (No engagement or team working skills) (0)	Below standard (No engagement) (0)
Total marks - /10									
