# Supplementary material for: Learner agency in a problem-based learning curriculum: A qualitative study on perspectives of undergraduate dental students
Source: PLoS One. 2026 Mar 26;21(3):e0346079. doi: 10.1371/journal.pone.0346079 (PMC13020853; doi:10.1371/journal.pone.0346079)
Supplement: S3 Appendix — (DOCX) [file pone.0346079.s003.docx]

**S2 Appendix: Topic Guide**

|  | **Questions included in the topic guide for the focus group** |
| --- | --- |
| **Past Experiences**   \| *(Exploring prior education and motivations for choosing dentistry)* \| \| --- \|  \|  \| \| --- \| | **Q1:** Can you describe your schooling experience before joining this program? *(Probes: What type of schools did you attend? Teaching methods?)*  **Q2:** What motivated you to pursue dentistry? *(Probes: Was there any family influence or personal inspiration?)* |
| **Present Experiences**  *(Understanding engagement, confidence, learning strategies, and external support based on the three dimensions)* | **Dimensions** |
|  | 1. **Intrapersonal** (Personal beliefs, values, motivation, and self-efficacy)   **Q3:** What aspects of your current studies do you find most engaging? *(Probes: Content, future career prospects, PBL approach, specific teachers?)*  **Q4:** In what ways do you feel confident in your journey to becoming a dentist? What factors contribute to this confidence? *(Probes: Good grades, participation in PBL discussions, independent learning, hands-on skills, communication?)* |
|  | 1. **Behavioral** (Actions, strategies, self-regulation, and reflection)   **Q5:** What are your individual and team-based learning goals? How do you plan and structure your studies?  **Q6:** What challenges or unexpected situations do you face in your learning, and how do you handle them?  **Q7:** How do you evaluate your contributions within your team? How do you and your peers encourage self-reflection? |
|  | 1. **Contextual** (Learning environment, resources, and social interactions)   **Q8:** How do you collaborate with your peers in team-based learning? What actions do you take to contribute to teamwork?  **Q9:** What types of support do you receive from your teachers and institution? *(Probes: Feedback, mentoring, access to resources?)* |
| \| **Future Perspectives**  *(Understanding perceptions of professional preparedness)* \| \| --- \| | **Q10:** How do you think your current learning experiences will help you in your future career as a dentist? *(Probes: Professional skills, confidence, real-world preparedness?)* |
