# Supplementary material for: Learner agency in a problem-based learning curriculum: A qualitative study on perspectives of undergraduate dental students
Source: PLoS One. 2026 Mar 26;21(3):e0346079. doi: 10.1371/journal.pone.0346079 (PMC13020853; doi:10.1371/journal.pone.0346079)
Supplement: S4 Appendix — (DOCX) [file pone.0346079.s004.docx]

**S4 Appendix: Coding scheme with sample themes and data.**

| Dimensions | Sub theme from the framework (deductive) | Sub theme from the bottom-up analysis (inductive) | Samples |
| --- | --- | --- | --- |
| Intrapersonal | Self-efficacy |  | *“With PBL, we research and connect ideas ourselves, which builds resilience for handling unexpected queries.” (FG1; participant 2)* |
|  | Motivation |  | *“With PBL, it feels like I’m learning for myself rather than just for an assessment which really motivates me.” (FG4; participant 6)* |
|  | Knowledge Acquisition |  | *“Patients have complex, multi-faceted issues, and PBL prepares us to integrate our knowledge to meet those varied needs”. (FG2; participant 3)* |
|  |  | Professional skills (Presentation and communication) | *“As future dentists, we’ll need to present patient cases, progress, and prognosis concisely. PBL helps us practice organizing and sharing information effectively, which also strengthens our communication skills.” (FG2; participant 2)* |
|  |  | Career perspective | *“PBL contributes to my future profession as a dentist. However, I wish it were more dentistry focused.” (FG1; participant 1)* |
| Behavioral | Self-regulated learning (Goal setting, planning and monitoring, reflection) |  | *“PBL helps us collaborate by making us set learning objectives, divide tasks, and communicate our findings. It also helps us understand different perspectives, which is essential for working with diverse patients and colleagues.” (FG3; participant 4)* |
| Contextual | Support from peers, teachers, institution | Resource accessibility  Educational Pressure | "*I think PBL provides a good balance each week. Instead of just having ten lectures, we get a mix of five lectures, PBL sessions, and SDL.” (FG2; participant 5)* |
